# Antimicrobial Activity of 2-(Piperazin-1-yl)naphtho[2,3-d]thiazole-4,9-dione against Staphylococcus Strains

**DOI:** 10.3390/molecules29061277

**Published:** 2024-03-13

**Authors:** Tamami Haraguchi, Saki Hayashi, Seira Nakasaka, Yoshiro Hatanaka, Toshihiro Nagao, Shigemitsu Tanaka, Miki Yoshii, Fumiko Hara, Masayori Hagimori, Miyako Yoshida

**Affiliations:** 1 Department of Clinical Pharmaceutics, Faculty of Pharmaceutical Sciences, Mukogawa Women’s University, 11-68 Koshien 9-Bancho, Nishinomiya 663-8179, Hyogo, Japan; tsuchiko_tamami_x@mukogawa-u.ac.jp (T.H.); hayashi_saki_x@mukogawa-u.ac.jp (S.H.); 1913743@mwu.jp (S.N.); 2 Institute for Women’s Career Advancement and Gender Equality Development, Mukogawa Women’s University, 6-46 Ikebiraki, Nishinomiya 663-8558, Hyogo, Japan; 3 Osaka Research Institute of Industrial Science and Technology, 1-6-50 Morinomiya, Joto-ku, Osaka 536-8553, Osaka, Japan; hatanaka@orist.jp (Y.H.); nagao@orist.jp (T.N.); s-tanaka@orist.jp (S.T.); yoshii@orist.jp (M.Y.); 4 Department of Analitical Chemistry, Faculty of Pharmaceutical Sciences, Mukogawa Women’s University, 11-68 Koshien 9-Bancho, Nishinomiya 663-8179, Hyogo, Japan; fhara@mukogawa-u.ac.jp

**Keywords:** 2-(piperazin-1-yl)naphtho[2,3-d]thiazole-4,9-dione, transmission electron microscopy, antimicrobial, DNA gyrase, fluorescence

## Abstract

There is an urgent need to discover and develop novel antibacterial agents. Accordingly, we synthesised 2-(piperazin-1-yl)naphtho[2,3-d]thiazole-4,9-dione (PNT), which exhibits antimicrobial activity. The aim of this study was to characterise PNT as an effective antimicrobial agent. Fluorescence microscopy was used to measure PNT’s uptake into microbial cells (strains of *Staphylococcus epidermidis*, *Staphylococcus aureus*, and methicillin-resistant *S. aureus* (MRSA)), transmission electron microscopy (TEM) was used to investigate the influence of PNT on the configuration of microbial cells, and a DNA gyrase supercoiling assay was used to investigate whether PNT inhibits DNA gyrase. PNT was taken up by more than 50% of microbial cells within 30 min. Using TEM, hollowed-out bacterial cytoplasms were observed in the specimen treated with PNT, although there was no disintegration of the bacterial membrane. In the DNA gyrase supercoiling assay, a dose-dependent reduction in fluorescence intensity was observed as the concentration of PNT increased. This suggests that PNT is taken up by microbial cells, resulting in cell disruption, and it reveals that one of the mechanisms underlying the antimicrobial activity of PNT is the inhibition of DNA gyrase.

## 1. Introduction

*Staphylococcus* species, including *S. epidermidis* and *S. aureus*, are Gram-positive cocci widely distributed in humans, mammals, and birds. *S. epidermidis* is the most universal commensal bacterial species in humans. In particular, *S. epidermidis* is the most frequently isolated of the three *Staphylococcus* species. It colonizes in the epithelia of the axillae, head, and nares predominantly. Through genome analysis, *S. epidermidis* is assumed to have genes to protect it from the harsh conditions of its natural habitat. *S. epidermidis* has some transport systems and some proton exchangers for osmoprotectants to cope with extreme of osmotic pressure and salt concentrations. *S. aureus* belongs to the group of coagulase-positive staphylococci. Meanwhile, *S. epidermidis* belongs to coagulase-negative staphylococci as it lacks the enzyme coagulase. However, in immunocompromised patients, it can be an opportunistic pathogen and may result in the development of severe infections [1,2]. Among the coagulase-negative staphylococci, *S. epidermidis* clearly causes the greatest number of infections. In clinical microbiology, coagulase-negative staphylococci are often not further specified, as the major interest is in distinguishing between *S. aureus* and other staphylococci. However, based on reports that have included species identification, one can assume that the vast majority of non-specified coagulase-negative staphylococci infections are due to *S. epidermidis*. In addition, *S. epidermidis* produces several factors used to colonise tissues or artificial surfaces, such as catheters [3]. Patients, especially those with an immunocompromised status, are susceptible to nosocomial infections through the use of devices and invasive procedures [4]. Biofilms are multicellular and surface-attached agglomerations of microorganisms. Biofilm, a structure formed by microorganisms, leads to the drug and host defence resistance of many antimicrobial agents. According to this general observation, *S. epidermidis* shows adaptation to a biofilm growth mode, including a down-regulation of nucleic acid, protein, and so on, in basic cell processes and cell wall biosynthesis especially. These gene regulatory changes may contribute to its drug resistance to many antimicrobial agents that target the actively growing cells of *S. epidermidis* biofilms.

*S. aureus* can live even in severely stressful environments because of its high adaptability. Therefore, it has been hypothesized to have regulatory systems to manage stress, such as the oxidative stress from superoxide radicals or hydrogen peroxide. The complexity and interrelationships of the response-to-stress systems with the systems involved in metal ion homeostasis are suggested in the regulatory systems that manage stress. However, the specific mechanism underlying the regulatory system that manages oxidative stress is unclear. Furthermore, reactive oxygen species (ROS) tend to operate in a lethal rather than in a bacteriostatic manner. ROS also have little effect on the minimum bactericidal concentration (MBC), which reinforces the point that ROS accelerate the lethal activity of antimicrobials. More research on precise stress responses to infectious conditions is needed.

*S. aureus* is the most pathogenic species belonging to this genus, and it causes suppurative disease or staphylococcal food poisoning. Moreover, it produces the enzyme coagulase, a catalysing protein that loosens the cellular basal membrane, thus disrupting the interstitial layers of the tissue, causing a wider spread of the infection [5,6].

One of the adaptabilities of *S. aureus* is it resistance to most usable antibiotics through its acquisition of conferring genes. The increased misuse of antimicrobial agents can result in the development of antibiotic resistance in *S. aureus*, including its resistance to methicillin, which leads to the emergence of methicillin-resistant *S. aureus* (MRSA) [7]. MRSA causes community- and healthcare-associated infections with higher rates of morbidity and mortality than those associated with methicillin-sensitive *S. aureus* [8]. The current antibiotics used for MRSA treatment include vancomycin, daptomycin, and linezolid; however, resistance to all three has emerged [9,10,11,12]. *S. aureus* with a reduced susceptibility to vancomycin and teicoplanin is suggested to be associated with a significant thickening of the cell wall by studies which have been conducted in many countries. However, the relationship between the antibiotic susceptibility and cell wall thickness of *S. aureus* is not clear. A part of the site-specific mobile chromosome cassette, *mecA,* in *S. hominis* has been suggested to have been transferred from an MRSA to a previously methicillin-susceptible strain present in a microbial community. Owing to the spread of multidrug-resistant bacteria such as MRSA, the current arsenal of effective antibiotics is being rapidly depleted. Therefore, there is an urgent need to identify and develop novel antibacterial agents.

Naphtho[2,3-d]thiazole-4,9-dione is a fused heterocyclic compound containing thiazole and naphthoquinone. The development of several naphtho[2,3-d]thiazole-4,9-dione derivatives with anticancer and antimicrobial activities has been reported, and N-(4,9-dioxo-4,9-dihydronaphtho[2,3-d]thiazol-2-yl)benzamide (TBA) is known to have antimicrobial activity [13]. Based on this, we synthesized 2-(piperazin-1-yl)naphtho[2,3-d]thiazole-4,9-dione (PNT), a novel compound showing antimicrobial activity. In this study, the antimicrobial activity of PNT against strains of *S. epidermidis*, *S. aureus*, and MRSA and its underlying mechanisms were investigated.

## 2. Results and Discussion

PNT was synthesized from the reaction of 2-(methylsulfinyl)naphtho[2,3-d]thiazole-4,9-dione and piperazine, with a 50% yield. TBA is known to have antimicrobial activity. The structures and calculated physical properties of both TBA and our novel compound, PNT, are listed in Table 1. PNT had a lower logD and higher logS. Thus, PNT has hydrophilic properties and a higher solubility than TBA. The MICs of TBA and PNT were also determined. The MICs of TBA against *S. epidermidis*, *S. aureus*, and MRSA were all 40 ± 0 μg/mL. Based on a relative method, the MICs of PNT against *S. epidermidis*, *S. aureus*, and MRSA were 2.5 ± 2.2 μg/mL, 2.5 ± 0.0 μg/mL, and 6.7 ± 2.9 μg/mL, respectively (Table 2). These results suggest that the antimicrobial activity of PNT against *S. epidermidis*, *S. aureus*, and MRSA was higher than that of TBA. From the microspecies distribution simulation obtained using MarvinSketch, in water, 97% of PNT was determined to exist in a positive ionic form, whereas 100% of TBA was suggested to exist in an unelectrified molecular form, when the pH is 7. The positive ionic form of PNT can easily enter microbial cells because the cell walls of microbes generally have a net negative charge, resulting in its effective antimicrobial activity. Therefore, PNT may potentially be a useful antimicrobial compound with high efficacy. Furthermore, the MBCs of PNT against *S. epidermidis*, *S. aureus*, and MRSA were measured and calculated as 1.25 μg/mL, 5.0 μg/mL, and 10 μg/mL, respectively (Table 3). These MBCs were all within 2 or 0.5 of PNT’s MIC value; thus, its MIC and MBC values were almost identical because PNT was serially diluted two-fold in the assay. These results suggest that the antimicrobial activity of PNT is not only bacteriostatic but also bactericidal.

**Figure 1:**
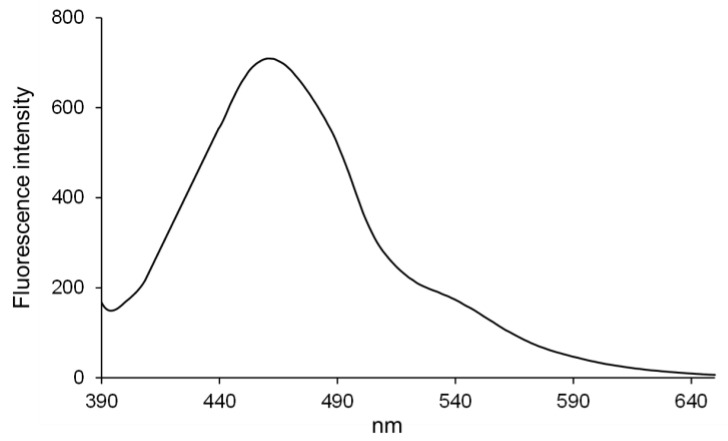
Fluorescence spectrum of PNT in H_2_O (excitation wavelength: 372 nm). PNT, 2-(piperazin-1-yl)naphtho[2,3-d]thiazole-4,9-dione.

PNT also exhibited fluorescence in an aqueous solution. The fluorescence spectrum of PNT in H_2_O is shown in Figure 1 (excitation wavelength: 372 nm). Its fluorescence intensity increased at an emission wavelength of 440–490 nm and its maximum fluorescence wavelength was 461 nm. In addition, PNT showed very large Stokes shifts (approximately 90 nm), which is advantageous for its detection without much interference from the excitation light. These results indicated that PNT could be used for fluorescence imaging. Then, fluorescence microscopy was further used to measure the uptake of PNT into the cells of three staphylococcus strains (*S. epidermidis*, *S. aureus*, and MRSA).

**Figure 2:**
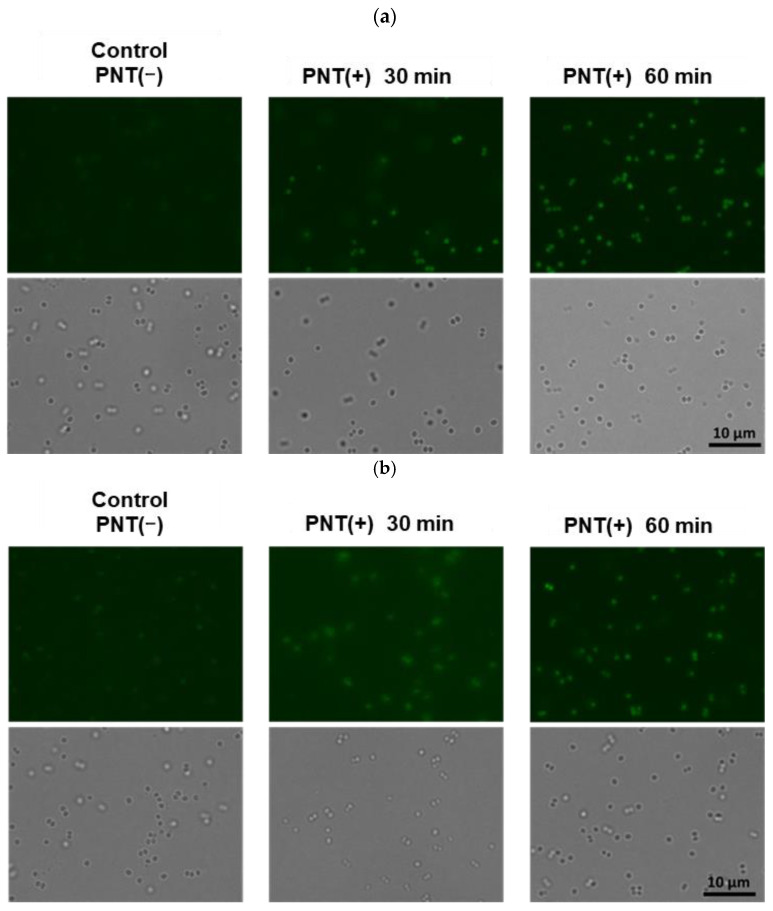

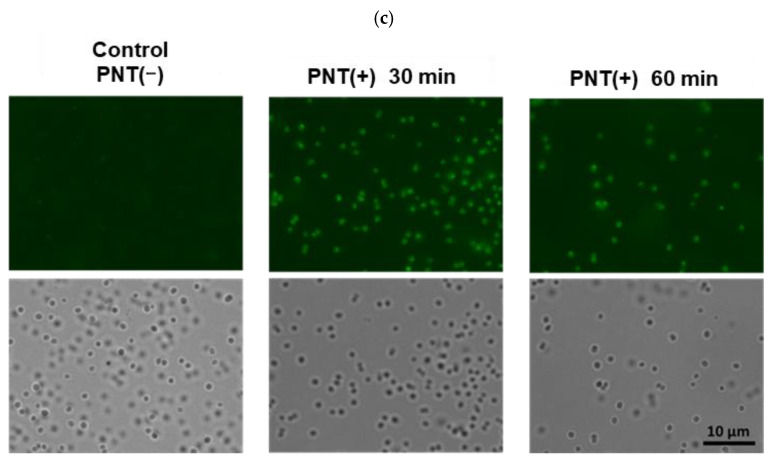
Uptake of PNT into *S. aureus* (**a**), methicillin-resistant *S. aureus* (MRSA) (**b**), and *S. epidermidis* (**c**), as observed via fluorescence microscopy. PNT, 2-(piperazin-1-yl)naphtho[2,3-d]thiazole-4,9-dione.

**Figure 3:**
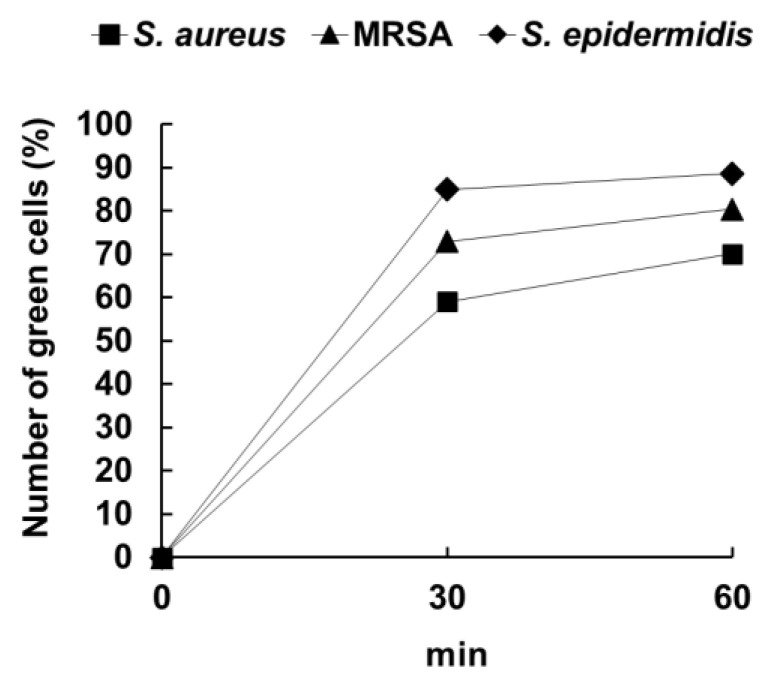
Percentages of green-fluorescing cells, indicating the uptake of PNT. PNT, 2-(piperazin-1-yl)naphtho[2,3-d]thiazole-4,9-dione.

**Figure 4:**
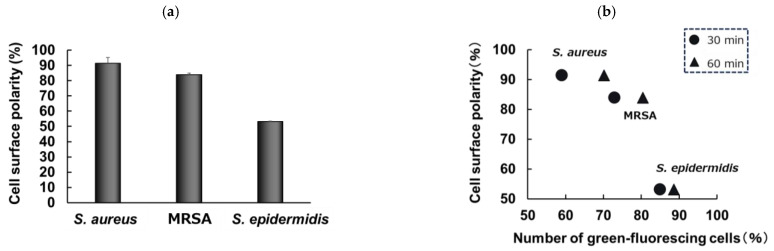
Cell surface polarity (**a**) and the relationship between the percentages of green-fluorescing cells, indicating the uptake of PNT, and surface polarities of each microbial strain (**b**). PNT, 2-(piperazin-1-yl)naphtho[2,3-d]thiazole-4,9-dione.

The fluorescence microscopy results, based on the observations of microbial cells treated with or without PNT, are shown in Figure 2. Many microbial cells treated with PNT exhibited green fluorescence after 30 and 60 min. To compare the ease of PNT’s uptake among the microbial strains, the number of green fluorescent cells was quantified. The percentage of green fluorescent cells was calculated from the total number of cells in each microphotograph. The calculated percentages of green-fluorescing cells, which indicated an uptake of PNT, are shown in Figure 3. At 30 and 60 min, more than 50% of cells were green-fluorescing across all strains. Further, *S. epidermidis* showed the highest rate of green fluorescence, whereas *S. aureus* showed the lowest. These results suggest that PNT is taken up more easily by *S. epidermidis* than by *S. aureus*. Owing to the presence of both hydrophilic and hydrophobic moieties, microbial surfaces are complex [14]. The hydrophilic properties of the cell wall are mainly attributed to charged groups, such as carboxyl, phosphate, amino, guanidyl, and dihydroxyl groups. In contrast, its hydrophobic properties are mostly attributed to the lipids and lipopolysaccharides present on the cell surface. The microbial cell wall has a net negative charge, and the magnitude of this charge can vary [15]. The surface polarities of the *S. epidermidis*, *S. aureus*, and MRSA used in this study were 53%, 91%, and 83%, green-fluorescing cells respectively (Figure 4a). Figure 4b shows the relationship between the percentage of and the surface polarities of the microbial strains. Here, *S. epidermidis*, which took up PNT more easily than the other strains, had the lowest surface polarity. It was suggested that the low surface polarity of microbial cells is important for their intracellular incorporation of PNT as an antimicrobial compound, whereas the negative charge of the microbial cell wall is important for PNT bonding to microbial cells.

**Figure 5:**
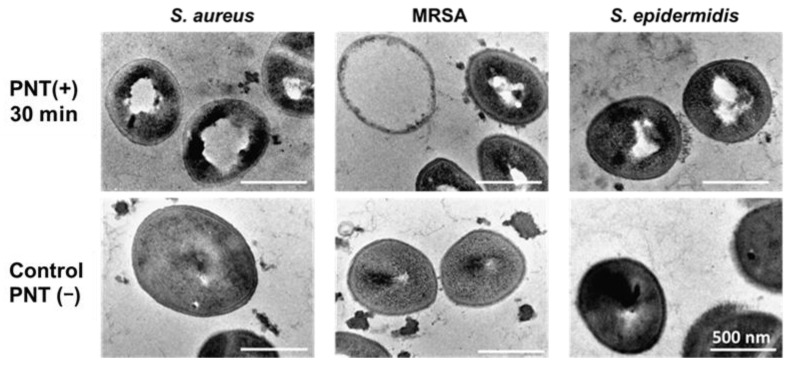
TEM images of microbial strains treated with or without PNT. TEM, transmission electron microscopy; PNT, 2-(piperazin-1-yl)naphtho[2,3-d]thiazole-4,9-dione.

The structure of each strain after taking up PNT was observed via TEM, which makes it possible to observe the insides and surfaces of cells [16,17,18,19,20,21]. The TEM images of the microbial strains treated with or without PNT are shown in Figure 5. The coccoidal shapes of *S. epidermidis*, *S. aureus*, and MRSA were preserved, and clear cell surfaces were observed for all specimens, with or without PNT. However, hollowed-out bacterial cytoplasms were observed in the specimens treated with PNT, although there was no disintegration of the bacterial membrane. These results suggest that PNT affects some cellular functions within the cell after it is taken up.

The chemical structure of PNT is similar to those of new quinolone-based antimicrobial agents, especially norfloxacin. Considering the TEM images of the microbial strains treated with or without PNT in this study, and the similarity of its chemical structure to these new quinolone-based antimicrobial agents, the target site of the antimicrobial activity of PNT against *S. epidermidis*, *S. aureus*, and MRSA was suggested to be the inner bacterial cell and not the bacterial cell membrane.

**Figure 6:**
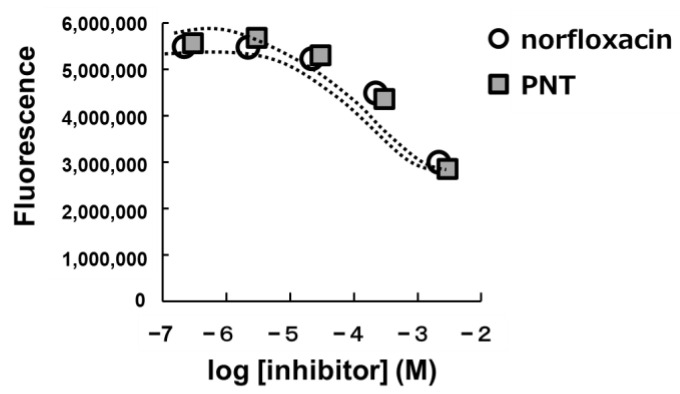
Dose–response curves plotting the fluorescence intensities of the DNA gyrase supercoiling assay.

New quinolone-based antimicrobial agents have a broad spectrum against both Gram-positive and -negative bacteria. They are especially used for the treatment of pathogenic microbial-caused respiratory system infections in several infectious diseases. In addition, the main mechanism through which they exert their antimicrobial activities is suggested to be the inhibition of microbial topoisomerases. Bacterial topoisomerases are also confirmed clinical targets for antimicrobial drug discovery. Bacterial topoisomerases, particularly DNA gyrase and topoisomerase IV, share high structural and sequence similarity but play distinct, required roles in DNA replication. In microbial cells, DNA gyrase catalyses the topological interconversions necessary for DNA’s replication, transcription, and recombination [22,23,24]. DNA gyrase inhibitors have a bactericidal effect by inhibiting nucleic acid synthesis, as described previously [25,26,27,28]. DNA gyrase inhibitors are similar to topoisomerase IV inhibitors in terms of their mechanism of inhibition of the enzyme’s catalytic activity. DNA gyrase’s catalytic activity involves the transduction of energy from adenosine triphosphate (ATP) hydrolysis and driving the passage of one intact DNA strand after two DNA strands become loose, leading to its transient transfer to the tyrosine residues on DNA gyrase. Both DNA gyrase and topoisomerase IV inhibitors block ATP binding and consequently interfere with DNA binding or DNA strands’ passage. DNA gyrase inhibitors have the high possibility of exerting this activity against topoisomerase IV due to the similarity between their targets. This dual targeting is suggested to lead to low mutation frequencies for drug resistance. This is one of the valuable features of the DNA gyrase inhibitors. Ciprofloxacin, one of the new quinolone-based antimicrobial agents, primarily targets the DNA gyrase or topoisomerase IV enzymes in bacteria, firstly to stabilize the cleavage DNA complex. Secondly, it blocks DNA replication and finally leads to microbial cell death. There is an Arg456 residue of topoisomerase IV near the C7 site of quinolone which is located in the topoisomerase IV–DNA complex, as suggested by its structure–activity relationship. The mutation of the Arg456 residue in the topoisomerase IV enzyme is suggested to cause quinolone resistance. Furthermore, substitutions of fluoroquinolones at position C7 significantly affect the cell’s permeability, which has an effect on its pharmacokinetic properties, as well as its bacterial resistance to ciprofloxacin. Therefore, the piperazinyl C-7 position of ciprofloxacin has the possibility to be used to develop new antimicrobial agents with a broad antimicrobial spectrum that can overcome drug resistance. Similar to ciprofloxacin, norfloxacin is also a new quinolone-based antimicrobial agent (Figure S1). It is a DNA gyrase inhibitor, bearing a piperazine ring, and PNT has the same structure [29,30,31,32]. Therefore, we focused on DNA gyrase inhibition when considering the mechanism underlying the antimicrobial effects of PNT. To determine whether PNT inhibits DNA gyrase, similar to norfloxacin, a DNA gyrase supercoiling assay was performed. The supercoiling mechanism facilitates the conversion of relaxed circular DNA into supercoiled DNA [28]. In the case of DNA interacting with the fluorescent dye H19, DNA yields different fluorescent intensities depending on its structure. The fluorescence signal in the supercoiled form of DNA potently increases compared with that of relaxed DNA. The change in the fluorescence intensity of DNA is used for the determination of a gyrase supercoiling reaction and to perform high-throughput screens for gyrase inhibitors [28]. In this study, a DNA supercoiling assay was conducted using PNT. A dose-dependent reduction in fluorescence intensity was observed as the concentration of PNT was increased. Norfloxacin served as a positive control DNA gyrase inhibitor. The dose–response curves plotting the fluorescence intensities of the DNA gyrase supercoiling assay are shown in Figure 6. The response curve for PNT was similar to that for norfloxacin. Moreover, PNT and norfloxacin exhibited half-maximal inhibitory concentration (IC_50_) values of 111.3 μg/mL and 94.1 μg/mL, respectively, against *S. aureus*-derived DNA gyrase. These results indicate that PNT inhibits DNA gyrase.

The binding affinity of antimicrobial agents and microbial cells vary based on their microbial species, which has specific amino acid sequences of DNA gyrase. In this study, *Staphylococcus* species were used. Additional experiments on other species of microbial strains would lead the way towards proper use of PNT as an effective antimicrobial compound. Quinolones are known to inhibit not only DNA gyrase but also topoisomerase IV. Further studies assessing the mechanism of antimicrobial activity of PNT, such as its inhibition of topoisomerase IV, would be useful for revealing the antimicrobial capability of PNT.

Recently, antibacterial lethality has focused on the contributsdion of reactive oxygen species (ROS) to lethal antimicrobial activity. Various antimicrobial agents are thought to create distinctive lesions that block growth and prompt a lethal stress response that culminates in an ROS cascade. The lethal action of some antimicrobial agents (fluoroquinolones, β-lactams, and aminoglycosides) is suggested to have relationship with ROS accumulation, because some agents and genes are thought to interfere with ROS’s accumulation and the lethal action of those antimicrobial agents. Members of the genetic pathways leading to a surge in ROS are reported to contribute to microbial lethality [33]. Resveratrol, an antioxidant, is known to interfere with the lethal action of ciprofloxacin on microorganisms. These data clearly support the hypothesis that ciprofloxacin generates ROS–antimicrobial lethality. PNT, with a chemical structure similar to ciprofloxacin, may also generate ROS–antimicrobial lethality.

A new series of ciprofloxacin–uracil conjugates formed by fusing the fluoroquinolone pharmacophore with uracil derivatives through an acetamide linker are reported to exhibit potent antibacterial activity against *S. aureus* and MRSA. Ciprofloxacin–uracil conjugates are reportedly more potent than their parent drug, ciprofloxacin. In addition, in comparison to ciprofloxacin, they also exhibit potent activities against MRSA. Moreover, ciprofloxacin–uracil conjugates show potent inhibitory activities against DNA gyrase and topoisomerase IV compared to ciprofloxacin. Molecular docking studies have revealed that ciprofloxacin–uracil conjugates may form stable interactions with the active sites of DNA gyrase and topoisomerase IV, similar to ciprofloxacin [34]. Hence, a PNT–uracil conjugate is expected to show potent inhibitory activity against DNA gyrase and topoisomerase IV compared to PNT, and potent antimicrobial activities against *S. aureus* and MRSA. From our results, the low surface polarity of microbial cells is suggested to be important for the intracellular incorporation of antimicrobial compounds, whereas the negative charge of the microbial cell wall is also suggested to be important for an antimicrobial compound bonding to microbial cells. The balance of these two properties, i.e., the polarity and hydrophobicity of the PNT–uracil conjugate, is suggested to be the key to the determination of its antimicrobial activity against *S. aureus* and MRSA.

## 3. Materials and Methods

### 3.1. Materials

All chemicals and reagents were obtained from commercial sources and used without further purification unless otherwise specified. The three microbial strains were purchased from the Biological Resource Center, National Institute of Technology and Evaluation (NBRC) (Tokyo, Japan), and the Japan Collection of Microorganisms (JCM) at the Riken BioResource Research Center (Ibaragi, Japan). The strains used in this study were *S. aureus* (NRBC 12732), MRSA (JCM 16555), and *S. epidermidis* (NBRC 100911).

### 3.2. Synthesis of 2-(Piperazin-1-yl)naphtho[2,3-d]thiazole-4,9-dione (PNT)

This compound was synthesized from the reaction of 2-(methylsulfinyl)naphtho[2,3-d]thiazole-4,9-dione (0.26 g, 1.0 mmol) and piperazine (0.17 g, 2.0 mmol) at 100 °C for 2 h. After cooling, 10 mL of methanol was added to the reaction mixture. The residue was collected through filtration. Recrystallisation from methanol yielded PNT, at a 50% yield (0.26 g, 1.0 mmol), in the form of orange needles. Its identification and measurements were performed using the following equipment. Its melting points were measured using a Yanako MP-500D micro melting point apparatus and are uncorrected. 1H and 13C-NMR spectra were obtained using the JEOL-JNM-EPC-400 (400 MHz) and JEOL-JNM-EPC-500 (500 MHz) spectrometers. Mass spectra (MS) were recorded using a JEOL MS-DX303 mass spectrometer, with the following conditions: Mp, 214–215 °C; 1H-NMR (DMSO-d_6_, 400 MHz) δ, 2.87 (4H, t, *J* = 5.2 Hz), 3.62 (4H, t, *J* = 4.8 Hz), 7.82–7.86 (2H, m), 7.99–8.07 (2H, m); 13C-NMR δ, (DMSO-d_6_, 125 MHz) δ 44.5, 49.5, 125.5, 126.6, 130.4, 131.9, 132.7, 133.5, 133.9, 154.1, 173.1, 176.6, 177.6 (Figures S2 and S3); MS m/z, 299 [M^+^]; HRMS calculated for C_15_H_13_N_3_O_2_S [M^+^], 299.0728; found, 299.0727.

### 3.3. Calculation of Physical Properties

A MarvinSketch (ChemAxon, Budapest, Hungary) was used to calculate the PNT’s logD (pH 7), logS (pH 7), polar surface area (pH 7), H-acceptor and H-donor (pH 7), pKa, polarizability, and hydrophilic–lipophilic balance (HLB).

### 3.4. Fluorescence Measurement of PNT

Fluorescence spectra were measured using a Jasco FP-8300 spectrometer. The evaluated compound was dissolved in DMSO to prepare a 10^−2^ mol/L solution. The sample solution was prepared by diluting the stock solution of PNT in H_2_O. The fluorescence intensities of the samples were measured at an excitation wavelength of 372 nm.

### 3.5. Determination of Minimum Inhibitory Concentrations (MICs) and Minimum Bactericidal Concentrations (MBCs)

The antibacterial activity of PNT was characterised by determining its MIC and MBC. Compound solutions were prepared using DMSO, and the final concentrations were 0, 0.2, 0.3, 0.6, 1.3, 2.5, 5, 10, 20, 40, and 80 μg/mL. Bacterial strains (*S. aureus*, MRSA, and *S. epidermidis*) in their stationary growth phase were diluted to a concentration of 4 × 10^4^ CFU/mL using Mueller-Hinton broth, pH 7 (Becton Dickinson, Franklin Lakes, NJ, USA); 150 µL of the culture was then dispensed into each well of a 96-well microtitre plate. The plates were incubated at 37 °C for 48 h. Each analysis was performed in triplicate. The MIC was determined as the minimum compound concentration at which the well was clear, indicating that no bacterial growth had occurred. The MBC was measured after a determination of the MIC after seeding, based on the absence of bacterial growth in new culture medium and after incubation at 37 °C for 48 h.

### 3.6. Fluorescence Microscopy to Measure PNT’s Uptake into Microbial Cells

Microbial cells were grown in Mueller-Hinton broth to their stationary phase (1.0 × 10^8^ CFU/mL); then, 112 μL of culture was treated with 8 µL (0.26 mg/mL in DMSO) of PNT and incubated at 37 °C. The cells were collected via centrifugation at 15,000× *g* for 15 min at different intervals (30, 60, and 120 min). Microbial pellets were then suspended in 100 µL of saline. A fixed volume of the bacteria (50 µL) was placed on a sterile glass slide, covered with a glass coverslip, and imaged using a fluorescence microscope (BZ-X810, KEYENCE, Osaka, Japan) with excitation/emission wavelengths of 480/500 nm for PNT. The number of green fluorescent cells was quantified using ImageJ (National Institutes of Health, Bethesda, MD, USA).

### 3.7. Cell Surface Polarity of Microbial Strains

Cell surface polarity was measured using the method based on microbial adhesion to hydrocarbons [35]. Microbes were grown in Mueller–Hinton broth to stationary phase (1.0 × 10^8^ CFU/mL), and then, cells were collected via centrifugation at 15,000× *g* for 15 min. Microbial pellets were then suspended in 3 mL of saline, and the optical density (660 nm) was measured (a). Then, 25 µL of n-hexadecanes was added to those samples with vortex mixing. After letting stand for 15 min, the optical density (660 nm) of the aqueous layer was measured (b). Cell surface polarity was calculated as follows: (a)/(b) × 100 (%).

### 3.8. Transmission Electron Microscopy (TEM)

Microbial cells were grown in Mueller-Hinton broth to their stationary phase (1.0 × 10^8^ CFU/mL), and then 45 μL of the culture was treated with 9 μL of PNT (0.4 mg/1.5 mL) for 30 min at 37 °C. After treatment, glutaraldehyde was added (final concentration 2.5%). The cells were centrifuged, and the pellets were embedded in 3% low-melting-point agar. The cells were then post-fixed in 4% buffered osmium tetroxide for 1 h, washed three times with ethanol, and embedded in epoxy resin (Quetol 651 Mix). Ultrathin sections (100 nm thick) were prepared using an ultramicrotome and mounted on a copper mesh. Finally, specimens were counterstained with 2% (*w*/*v*) uranyl acetate solution for 20 min and then with Reynolds lead solution for 5 min and examined using a JEM2100 transmission electron microscope (JEOL Ltd., Tokyo, Japan) operated at 200 kV.

### 3.9. DNA Gyrase Supercoiling Assay

An *S. aureus* Gyrase DNA Supercoiling Assay Kit Plus (ProFoldin) was used to measure the inhibitory effect of PNT on DNA gyrase. Each reaction mixture (total volume, 20 μL) was prepared and included 11.8 μL of PNT, or norfloxacin as a control DNA gyrase inhibitor; 2 μL of 10× buffer; 2 μL of 2 M potassium glutamate; 2 μL of 10× relaxed DNA (250 μg/mL); 0.2 μL of 7.5 μM *S. aureus* gyrase; and 2 μL of 10 mM ATP. The reaction mixture was then incubated at 37 °C for 90 min. At the end of the assay reaction, 100 μL of water was added. For the assay, 25 μL of H19 dye was mixed with 120 μL of the reaction solution in each well. The mixtures were then incubated at room temperature for 15 min. Their fluorescence intensities were measured at 530 nm using an excitation wavelength of 480 nm.

## 4. Conclusions

In this study, we synthesised PNT, which exhibited excitation fluorescence and antimicrobial activity. Our results suggest that PNT could be taken up by microbial cells, resulting in cell disruption, and reveal that one of the mechanisms underlying its antimicrobial activity is the inhibition of DNA gyrase. Further experiments to assess the other antimicrobial mechanisms of PNT or the designing of conjugates of PNT with other antimicrobial active compounds may reveal avenues for the proper use of PNT as an effective antimicrobial agent.

## Figures and Tables

**Table 1:** Structures and physical properties of TBA and PNT.

	TBA	PNT
	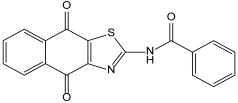	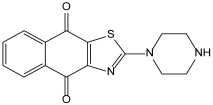
**logD**	3.87	0.83
**logS**	−6.46	−2.87
**Polarizability**	33.75	30.37
**HLB**	10.12	14.11
**Polar surface area**	104.37	95.12
**H-acceptor sites**	7	6
**H-donor sites**	1	2

HLB, hydrophilic–lipophilic balance; TBA, *N*-(4,9-dioxo-4,9-dihydronaphtho[2,3-d]thiazol-2-yl)benzamide; PNT, 2-(piperazin-1-yl)naphtho[2,3-d]thiazole-4,9-dione.

**Table 2:** MIC (µg/mL) of TBA and PNT against *S. epidermidis*, *S. aureus*, and MRSA.

	TBA	PNT
*S. epidermidis* (NBRC 100911)	40 ± 0	2.5 ± 2.2
*S. aureus* (NRBC 12732)	40 ± 0	2.5 ± 0.0
MRSA (JCM 16555)	40 ± 0	6.7 ± 2.9

**Table 3:** MBC (µg/mL) of TBA and PNT against *S. epidermidis*, *S. aureus*, and MRSA.

	PNT
*S. epidermidis* (NBRC 100911)	1.25
*S. aureus* (NRBC 12732)	5.0
MRSA (JCM 16555)	10

## Data Availability

The data presented in this study are available in article and Supplementary Materials.

## Supplementary Materials

The following supporting information can be downloaded at: https://www.mdpi.com/article/10.3390/molecules29061277/s1, Figure S1: Chemical structures of (A) ciprofloxacin and (B) norfloxacin; Figure S2: 1H NMR spectrum of PNT; Figure S3: 13C NMR spectrum of PNT.
