# Supplementary material for: Antimicrobial Activity of 2-(Piperazin-1-yl)naphtho[2,3-d]thiazole-4,9-dione against Staphylococcus Strains
Source: Molecules. 2024 Mar 13;29(6):1277. doi: 10.3390/molecules29061277 (PMC10975047; doi:10.3390/molecules29061277)
Supplement: Supplementary file 1 [file molecules-29-01277-s001.zip › molecules-2883474-supplementary.pdf]

Supplementary Materials

Antimicrobial Activity of 2-(Piperazin-1-yl)naphtho[2,3-d]thiazole-4,9-dione against Staphylococcus Strains

Tamami Haraguchi [1,2], Saki Hayashi [1], Seira Nakasaka [1], Yoshiro Hatanaka [3], Toshihiro Nagao [3], Shigemitsu Tanaka [3], Miki Yoshii [3], Fumiko Hara [4], Masayori Hagimori [4,*] and Miyako Yoshida [1,2,*]

[1] Department of Clinical Pharmaceutics, Faculty of Pharmaceutical Sciences, Mukogawa Women's University, 11-68 Koshien 9-Bancho, Nishinomiya 663-8179, Hyogo, Japan; tsuchiko_tamami_x@mukogawa-u.ac.jp (T.H.); hayashi_saki_x@mukogawa-u.ac.jp (S.H.); 1913743@mwu.jp (S.N.)

[2] Institute for Women's Career Advancement and Gender Equality Development, Mukogawa Women's University, 6-46 Ikebiraki, Nishinomiya 663-8558, Hyogo, Japan

[3] Osaka Research Institute of Industrial Science and Technology, 1-6-50 Morinomiya, Joto-ku, Osaka 536-8553, Osaka, Japan; hatanaka@orist.jp (Y.H.); nagao@orist.jp (T.N.); s-tanaka@orist.jp (S.T.); yoshii@orist.jp (M.Y.)

[4] Department of Analitical Chemistry, Faculty of Pharmaceutical Sciences, Mukogawa Women's University, 11-68 Koshien 9-Bancho, Nishinomiya 663-8179, Hyogo, Japan; fhara@mukogawa-u.ac.jp

* Correspondence: miyakoy@mukogawa-u.ac.jp (M.Y.); hagimori@mukogawa-u.ac.jp (M.H.); Tel.: +81-798-45-9965 (M.Y.); +81-798-45-9949 (M.H.)

Contents

1. Figure S1. Chemical structures of ciprofloxacin and norfloxacin

2. Figure S2. ^{1}H NMR spectrum of PNT

3. Figure S3. ^{13}C NMR spectrum of PNT

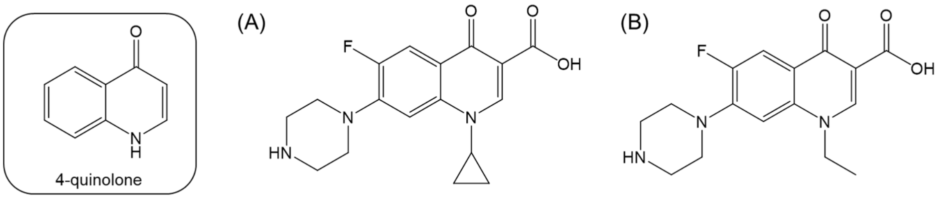

Figure S1. Chemical structures of (A) ciprofloxacin and (B) norfloxacin.

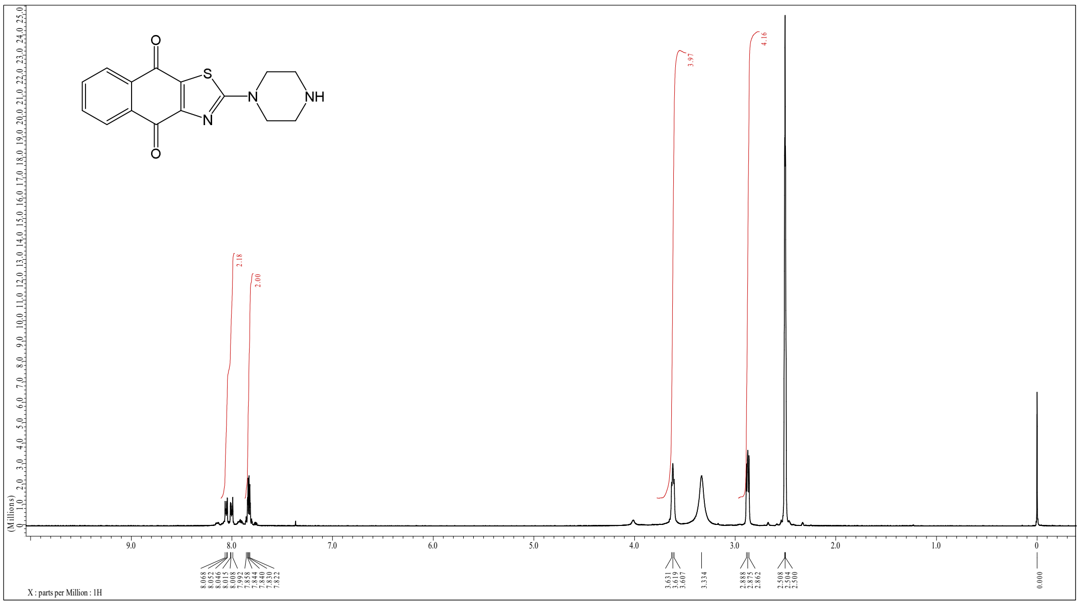

Figure S2. 1H NMR spectrum of PNT.

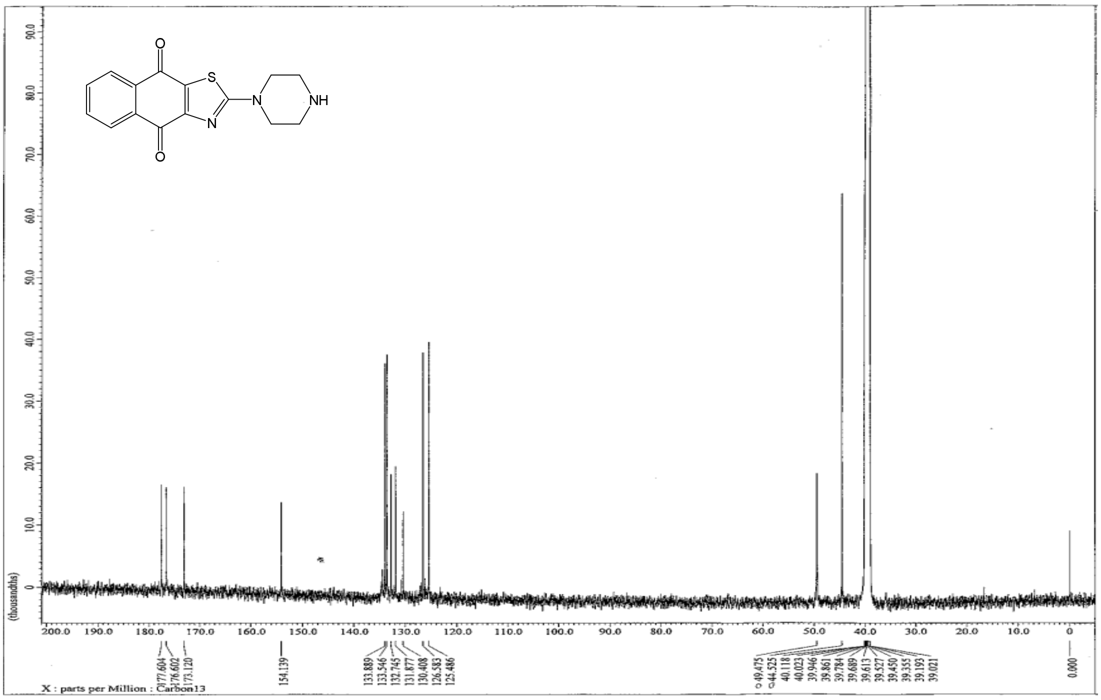

Figure S3. 13C NMR spectrum of PNT.
